# Improvement of FK506 production via metabolic engineering-guided combinational strategies in *Streptomyces tsukubaensis*

**DOI:** 10.1186/s12934-021-01660-w

**Published:** 2021-08-23

**Authors:** Qing-Bin Wu, Xiao-Ying Zhang, Xin-Ai Chen, Yong-Quan Li

**Affiliations:** 1 First Affiliated Hospital and Institute of Pharmaceutical Biotechnology, Zhejiang University School of Medicine , Hangzhou, 310058 China; 2 Zhejiang Provincial Key Lab for Microbial Biochemistry and Metabolic Engineering, Hangzhou, 310058 China

**Keywords:** *Streptomyces tsukubaensis*, FK506, Hyper-production, Metabolic engineering, Biosynthetic gene cluster refactoring, Regulatory circuits

## Abstract

**Background:**

FK506, a macrolide mainly with immunosuppressive activity, can be produced by various *Streptomyces* strains. However, one of the major challenges in the fermentation of FK506 is its insufficient production, resulting in high fermentation costs and environmental burdens. Herein, we tried to improve its production via metabolic engineering-guided combinational strategies in *Streptomyces tsukubaensis*.

**Results:**

First, basing on the genome sequencing and analysis, putative competitive pathways were deleted. A better parental strain L19-2 with increased FK506 production from 140.3 to 170.3 mg/L and a cleaner metabolic background was constructed. Subsequently, the FK506 biosynthetic gene cluster was refactored by in-situ promoter-substitution strategy basing on the regulatory circuits. This strategy enhanced transcription levels of the entire FK506 biosynthetic gene cluster in a fine-tuning manner and dramatically increased the FK506 production to 410.3 mg/mL, 1.41-fold higher than the parental strain L19-2 (170.3 mg/L). Finally, the FK506 production was further increased from 410.3 to 603 mg/L in shake-flask culture by adding L-isoleucine at a final concentration of 6 g/L. Moreover, the potential of FK506 production capacity was also evaluated in a 15-L fermenter, resulting in the FK506 production of 830.3 mg/L.

**Conclusion:**

From the aspects of competitive pathways, refactoring of the FK506 biosynthetic gene cluster and nutrients-addition, a strategy for hyper-production and potentially industrial application of FK506 was developed and a hyper-production strain L19-9 was constructed. The strategy presented here can be generally applicable to other *Streptomyces* for improvement of FK506 production and streamline hyper-production of other valuable secondary metabolites.

**Supplementary Information:**

The online version contains supplementary material available at 10.1186/s12934-021-01660-w.

## Background

FK506 (tacrolimus) is a 23-membered macrolide possessing immunosuppressive activities. Since its first discovery from *Streptomyces tsukubaensis* in 1987, it has been clinically used as an important immunosuppressant to prevent rejection of transplanted organs and it is approximately 100-fold more potent than the immunosuppressive compound cyclosporin A [1–6].

Characterization of the entire biosynthetic gene cluster for FK506 has facilitated genetic and biochemical studies on FK506 biosynthesis. The polyketide synthase (PKS) within the cluster incorporates the shikimate-derived 4,5-dihydroxycyclohex-1-enecarboxylic acid (DHCHC) as a starter unit and catalyzes ten successive polyketide chain elongation cycles with two malonyl-CoAs, two methoxymalonyl-ACPs, five methylmalonyl-CoAs and one allylmalonyl-CoA molecules [7, 8]. The linear polyketide chain was then condensed with pipecolate by the non-ribosomal peptide synthase (NRPS) FkbL and further modified by a series of post-PKS tailoring steps [9–11]. The genes *fkbG/H/I/J/K*, *tcsA/B/C/D* and *fkbL* within the cluster were responsible for biosynthesis of extender units methoxymalonyl-ACP, allylmalonyl-CoA and pipecolate, respectively [8, 12, 13].

Due to its valuable application, basing on random mutagenesis, medium optimization and genetic manipulation of specific gene targets, tremendous efforts have been dedicated to the enhancement of FK506 production. For example, FK506 production was improved by random mutagenesis in *Streptomyces* sp. KCCM 11116P [14]. Effect of genetic manipulation of the gene targets inside the FK506 biosynthetic pathway (including *fkbG/H/I/J/K*, *tcsA/B/C/D*, *fkbO*, *fkbL*, *fkbP*, *fkbM*, *fkbD*, *fkbN* and *tcs7*) [15–19] or outside the FK506 biosynthetic pathway (including *dahp*, *gdhA*, *accA2*, *zwf2*, *matB*, *mutAB* and *pcc*) [14, 20, 21] on FK506 production were also evaluated in related *Streptomyces* strains. Exogenous additives, such as carbon sources, amino acids, specific precursors, were also previously optimized/added to improve FK506 production [21–26]. However, some strategies were only conducted in strains with relatively low FK506 production or some strategies were too costly to use in industrial production. Taking both hyper-production and industrial application into consideration, how to construct a high-yielding strain for FK506 production rationally and systematically is of great importance and value.

**Fig. 1:**
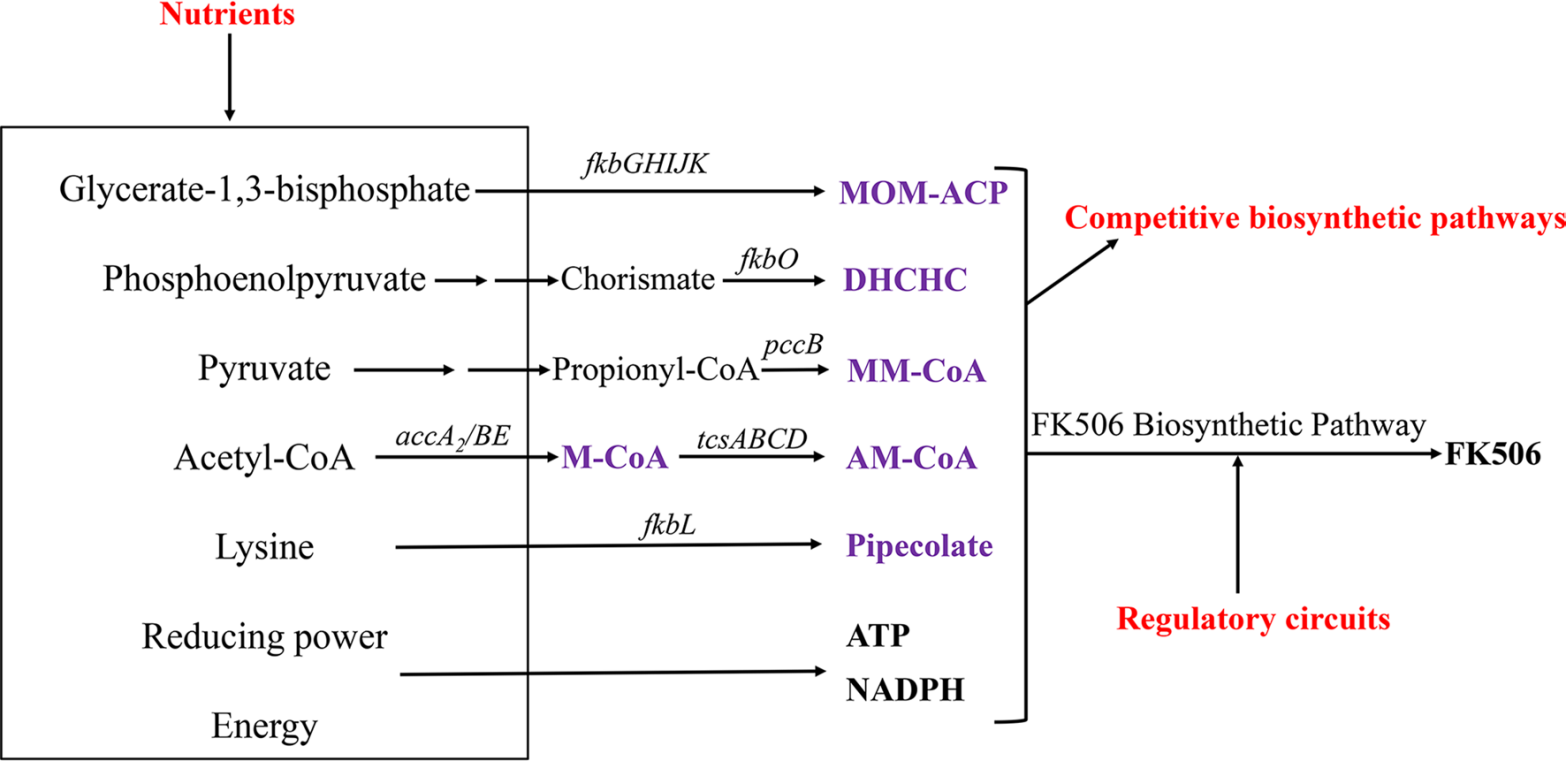
Schematic representation of metabolic pathways involved in FK506 biosynthesis. Precursors for FK506 biosynthesis were shown in purple. Targets for improved FK506 production were shown in red. Some important genes involved in the biosynthesis of relative precursors were also shown (italics)

In this study, basing on an understanding of the biosynthetic machinery, regulatory pathway related to FK506 biosynthesis and its rough metabolic fluxes in *S. tsukubaensis* L19 (Fig. 1), we developed a combinational strategy and constructed a FK506-hyper-production strain L19-9. The concepts of competitive pathways, biosynthetic gene cluster refactoring and nutrients supplementation applied in this work were generally applicable to and enable the production-improvement of other valuable natural products or FK506 in other *Streptomyces* strains.

## Results

### Genomic analysis of *S. tsukubaensis* L19

The genome sequence of *S. tsukubaensis* L19 had been determined by sequencing. The results demonstrated the L19 possessed a chromosome of 7,935,069 bp and had 7,142 predicted protein coding sequences.

Basing on the genome sequence, we performed analysis of secondary metabolite biosynthetic gene clusters with antiSMASH [27] in L19. The results showed that there are 35 putative biosynthetic gene clusters in the genome of *S. tsukubaensis* L19, 10 of which are PKS/PKS-NRPS gene clusters (Table 1).

**Table 1:** Putative PKS/PKS-NRPS gene clusters predicted in *S. tsukubaensis* L19

Cluster	Type	Length (bp)	Transcription
C1	Type I PKS	119,016	No
C2	Type I PKS	44,557	No
C3	Type I PKS	137,694	Yes
C4	Type I PKS/NRPS	74,351	No
C5	Type II PKS	86,334	No
C6	Type II PKS	72,521	Yes
C7	Type I PKS	42,869	No
C8	FK506 gene cluster	80,645	Yes
C9	Type I PKS	39,818	Yes
C10	Type I PKS	44,687	No

### Deletion of putatively competitive gene clusters for a better FK506-producing strain

Although FK506 is the product of a hybrid PKS-NRPS, only one molecule of lysine-derived pipecolate is needed as the extender unit for NRPS and ten molecules of acyl precursors (including two malonyl-CoAs, two methoxymalonyl-ACPs, five methylmalonyl-CoAs and one allylmalonyl-CoA) are needed as the extender units for PKS during biosynthesis of per molecule FK506. Hence, we mainly focused our attention on the putatively competitive PKS or PKS-NRPS gene clusters, which may compete for some common acyl precursors, such as malonyl-CoA. The total RNA was extracted from mycelia cultured 60 h and expression of the genes encoding core PKS located in these PKS/PKS-NRPS gene clusters was determined by reverse transcription polymerase chain reaction (RT-PCR). The results indicated that the genes encoding core PKS in clusters C3, C6 and C9 were all transcribed besides the FK506 gene cluster, which are supposed to compete with FK506 biosynthesis (Additional file 1: Fig. S1).

**Fig. 2:**
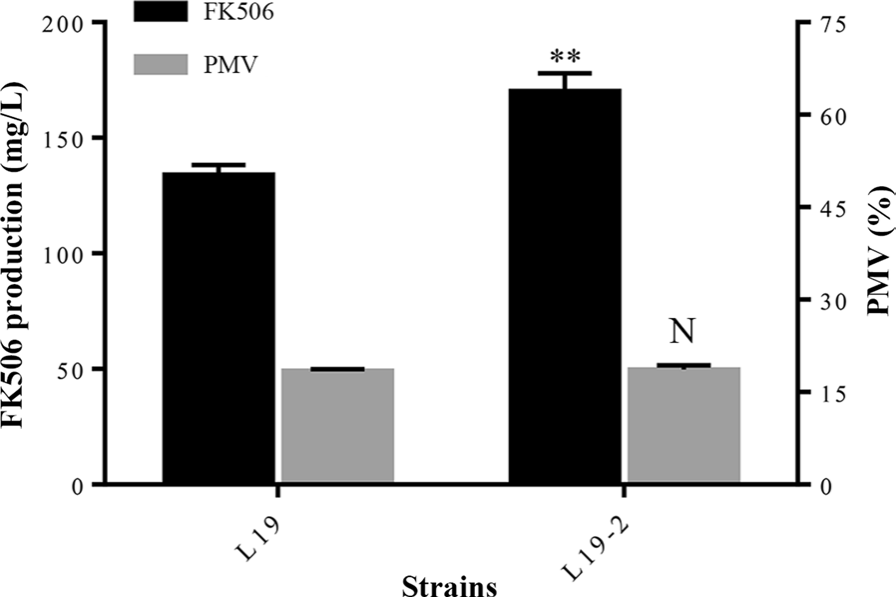
FK506 production and packed mycelium volume (PMV) in L19 and L19-2 at 168 h, respectively. Error bars showed standard deviations of three independent experiments. The asterisks indicated the statistic significant differences compared with L19, respectively. **P < 0.05; N, no significant difference

**Fig. 3:**
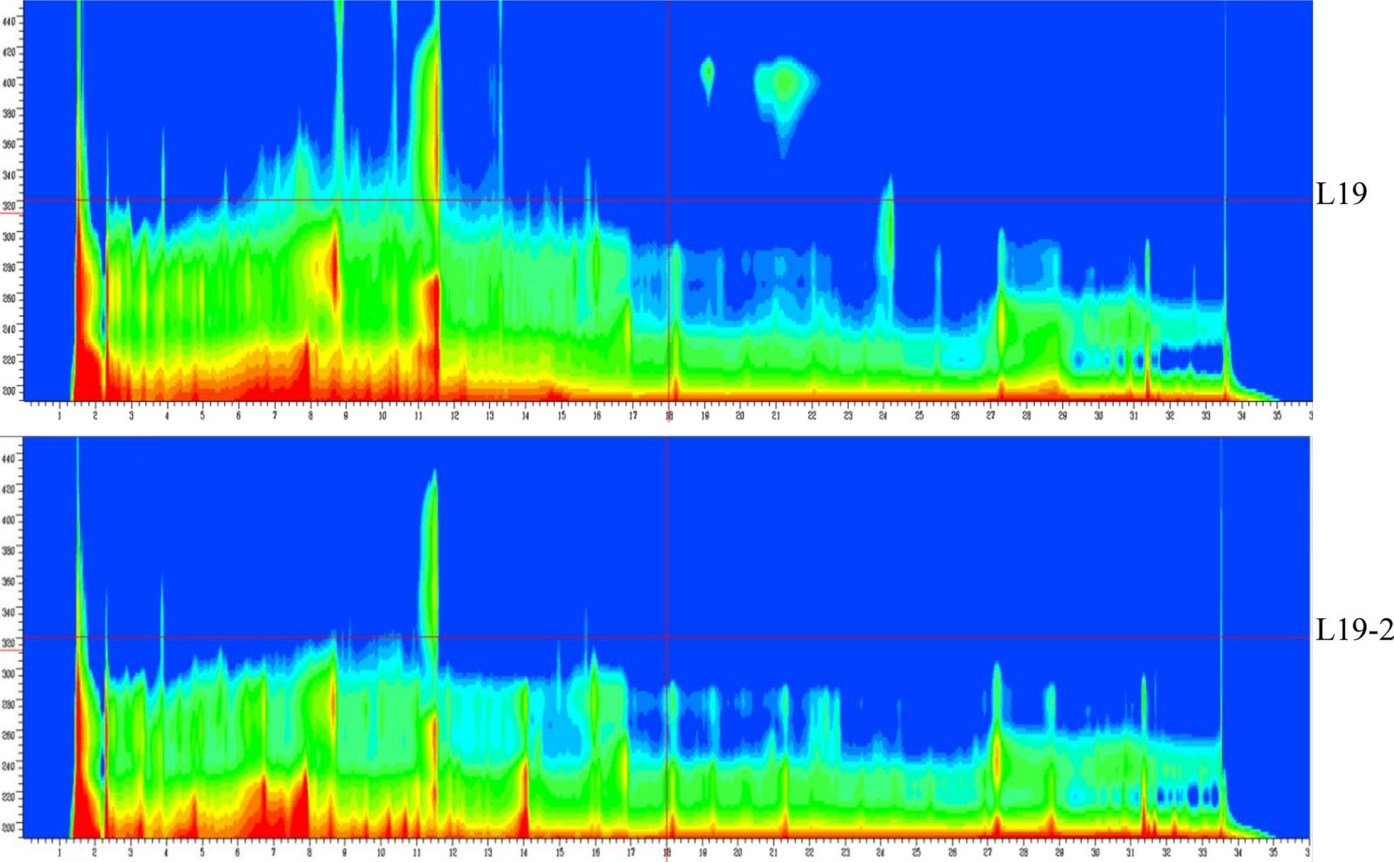
Analysis of metabolite profiles in L19 and L19-2 by full wavelength scanning. Equal volume of fermentation broths from different strains was harvested, ultrasonically extracted with 2.5-fold volume of methanol and then subjected to HPLC analysis. The wavelength scanned from 190 to 600 nm

Due to the difficulty in deleting large DNA fragments in the strain L19, only the genes encoding core PKS in these three clusters were deleted combinatorially in the parental strain L19 and the resultant mutant was named as L19-2. FK506 production of these strains were then monitored by HPLC analyses after fermentation for 168 h. As shown in Fig. 2**,** the production of FK506 in strain L19-2 increased from 140.3 to 170.3 mg/mL at 168 h, 21.4% higher than that in strain L19. Meanwhile, no significant difference in biomass was observed among the strains (Fig. 2**)**. Furthermore, by means of full wavelength scanning, we found that the resultant strain L19-2 possessed much cleaner metabolite profiles than L19 in Fm medium (Fig. 3). All these results indicated that the strain L19-2 was a better starting strain for production of FK506 compared with L19.

### Refactoring regulatory circuits in the FK506 biosynthetic pathway

**Fig. 4:**
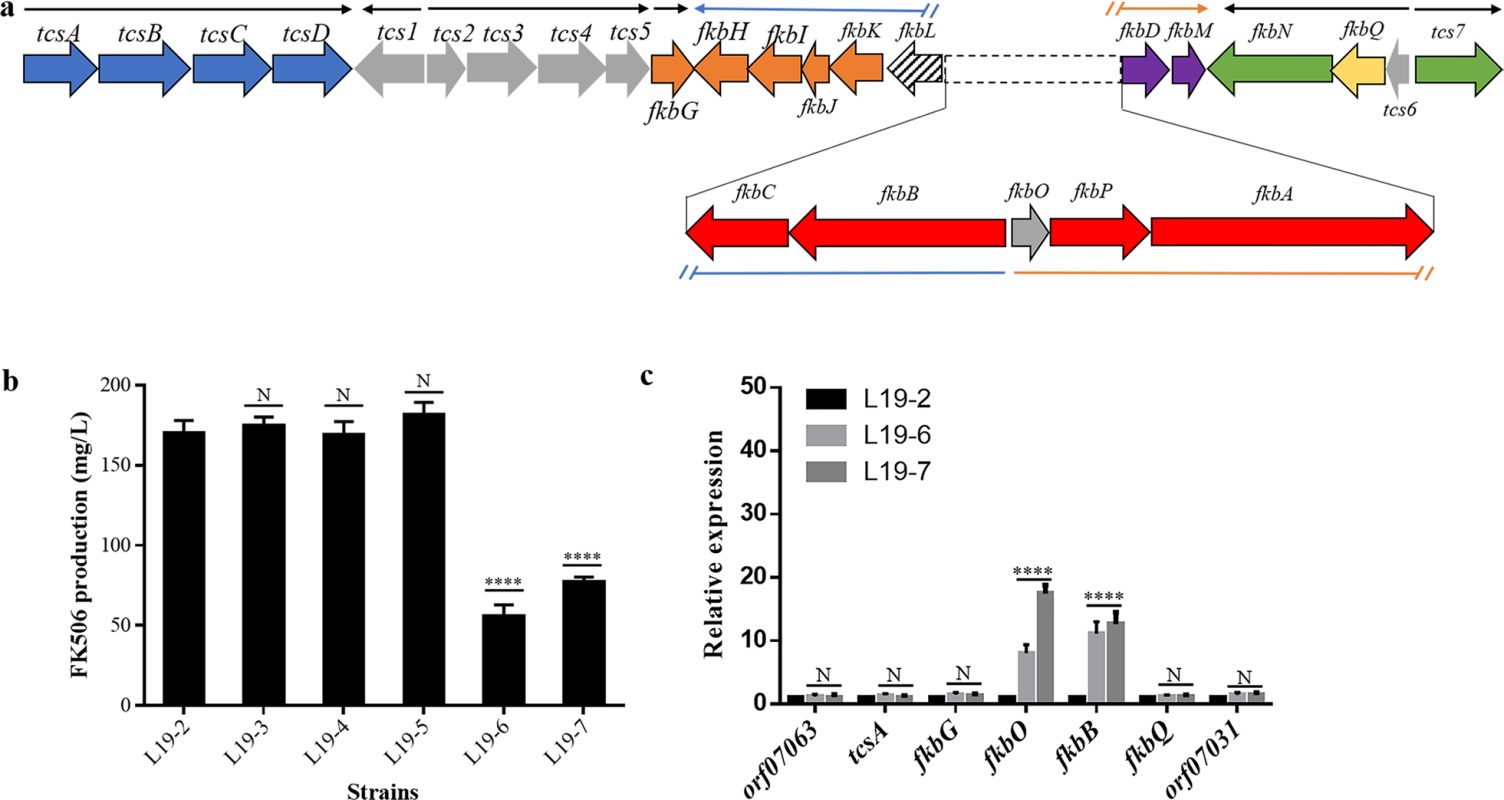
FK506 biosynthetic gene cluster and analysis of FK506 production and relative expression levels. **a** Proposed organization of the transcription units ( →) in FK506 biosynthetic gene cluster. **b** Effect on FK506 production by overexpressing *tcsA/B/C/D* (L19-3), *fkbG/H/I/J/K* (L19-4) or *fkbO* (L19-5) and in-situ substitution of the intergenic region between the gene *fkbB* and *fkbO* by constructed promoter cassettes (L19-6 and L19-7). **c** Relative expression levels of selected genes in *S. tsukubaensis* L19-6 and L19-7 compared with L19-2. Excluding genes located in the FK506 biosynthetic gene cluster, genes residing upstream and downstream regions of the FK506 biosynthetic gene cluster were also included. Each experiment was repeated for three times and error bars showed standard deviations. The asterisks indicated the statistic significant differences compared with L19-2, respectively. ****P < 0.0001; N, no significant difference

We had previously identified the FK506 biosynthetic gene cluster which is organized as eight transcription units in the strain L19 [17]. The transcription units involved in FK506 biosynthesis are *tcsA/B/C/D*, *fkbG*, *fkbB/C/L/K/J/I/H*, *fkbO/P/A/D/M*, *tcs6/fkbQ/N*, and *tcs7* (Fig. 4a)*.* To identify the rate-limiting steps, gene cassettes *tcsA/B/C/D* and *fkbG/H/I/J/K*, responsible for biosynthesis of the special extender units, were individually cloned into the integration vector pLM1 [28] to construct two plasmids pLM1-1 and pLM1-2, in which these genes were under control of the well-characterized promoter ermEp*. The plasmids were then individually introduced into L19-2 to construct the resultant strains L19-3 and L19-4, respectively. However, compared with the parental strain L19-2, the strains showed no further improvement in FK506 production (Fig. 4b). Subsequently, another reported gene *fkbO* responsible for biosynthesis of the starter unit DHCHC was also overexpressed (the resultant plasmid and strain named as pLM1-3 and L19-5, respectively), which surprisingly also showed no significant effect on FK506 production in the strain L19-2 (Fig. 4b).

**Fig. 5:**
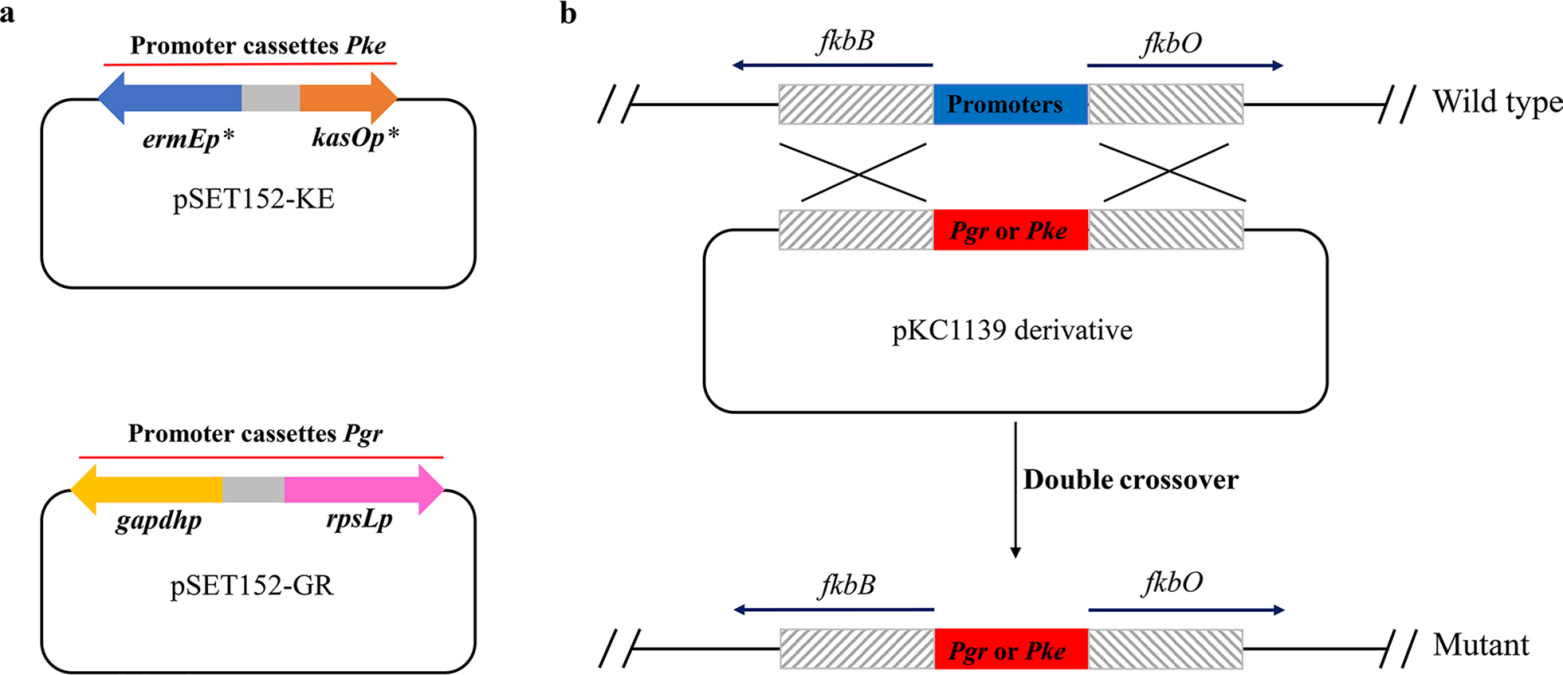
Schematic representation of the in-situ substitution strategy. **a** The bidirectional promoter cassettes *Pgr* and *Pke* were constructed based on the vector pSET152. The direction of the arrows represented the direction of the promoters. **b** Replacement of the intergenic region between *fkbB* and *fkbO* in situ by pKC1139-mediated double crossover

Considering that the remaining biosynthetic genes were mainly organized as two large transcription units (*fkbB/C/L/K/J/I/H* and *fkbO/P/A/D/M*) and their expression were under control of a bidirectional promoter (the intergenic region between *fkbB* and *fkbO*), we then tried to replace this region with stronger constitutive promoter cassettes. In our others work, we had found that the promoters *gapdhp* and *rpsLp* [29] possessing comparable activity were much stronger than that of the ermEp* [30] and kasOp* [31] promoters in strain L19. Hence, two bidirectional promoter cassettes *Pke* and *Pgr* were constructed and then used to replace the intergenic region between *fkbB* and *fkbO* in situ by pKC1139-mediated double crossover as shown in Fig. 5, respectively. Unexpectedly, the resultant strains (L19-6 and L19-7, respectively) produced even lower FK506 than the parental strain L19-2 (Fig. 4b), though transcriptional levels of related biosynthetic genes were increased compared with strain L19-2 (Fig. 4c). These results might suggest the importance of the transcriptional level balance between different operons within the gene cluster and we will further discuss this phenomenon in “Discussion” section.

In our previous work, we had verified that overexpression of the pathway-specific regulatory genes *fkbN* and *tcs7* could enhance the transcriptional levels of all biosynthetic genes [17]. Accordingly, we presumed that substitution of the intergenic region between *tcs6* and *tcs7* (a bidirectional promoter region controlling the expression of both *fkbN* and *tcs7*) with stronger constitutive promoter cassettes could indirectly enhance the transcription levels of all biosynthetic genes in a fine-tuning manner and thus improve FK506 production. Furthermore, comparing with overexpression-strategy basing on integrative vectors, this substitution strategy could prevent the introduction of additional resistance genes and the putative chromosomal position effect [32, 33], which made the resultant strain conducive for further genetic manipulation when needed.

**Fig. 6:**
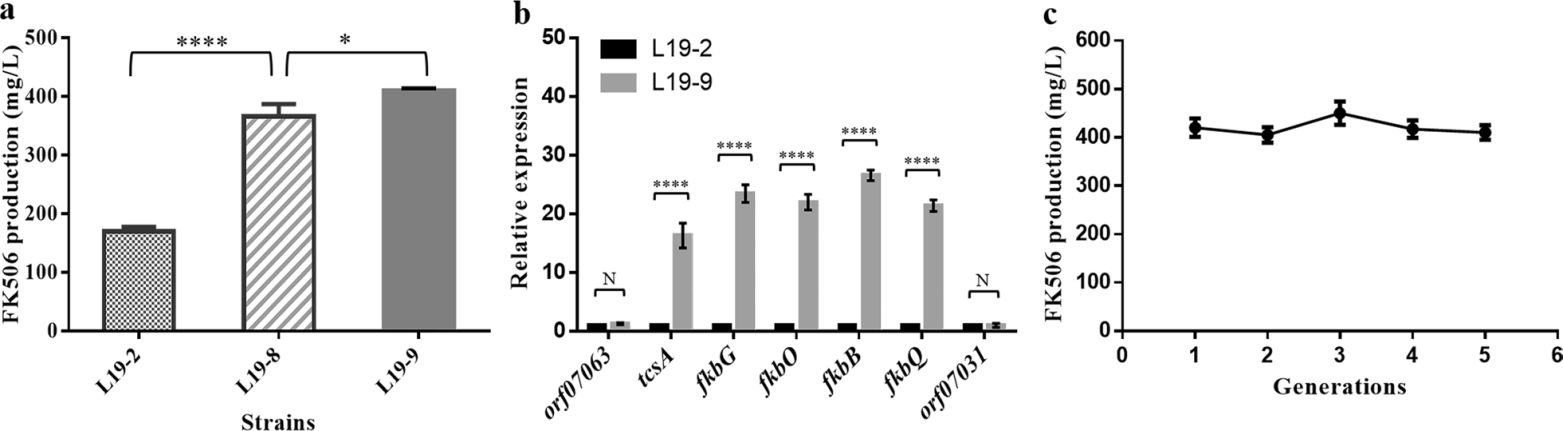
Analysis of FK506 production, relative expression levels and genetic stability. **a** FK506 production at 168 h in strains by substitution of the bidirectional promoter region controlling the expression of both *fkbN* and *tcs7.* **b** Relative expression levels of selected genes in *S. tsukubaensis* L19-9 compared with L19-2. Excluding genes located in the FK506 biosynthetic gene cluster, genes residing upstream and downstream regions of the FK506 biosynthetic gene cluster were also included. **c** Production-stability of the high-yielding strain L19-9 in shake-flask culture. The strain was continuously cultured for 5 generations on ISP4 agar plates and the FK506 production was analyzed. Each experiment was repeated for three times and error bars showed standard deviations. The asterisks indicated the statistic significant differences. *P < 0.05; ****P < 0.0001; N, no significant difference

The constructed bidirectional promoter cassettes *Pke* and *Pgr* were then used to replace the intergenic region between *tcs6* and *tcs7* in strain L19-2 as mentioned above, respectively. The resultant strains (designated as L19-8 and L19-9, respectively) were confirmed by PCR and further fermented for FK506 production analyses. As was expected, both L19-8 and L19-9 produced higher FK506 compared with L19-2 after fermentation for 168 h (L19-2, 170.3 mg/L; L19-7, 350 mg/L; L19-8, 410.3 mg/L) (Fig. 6a). Especially, the strain L19-9 possessing stronger promoters showed higher FK506 production than L19-8, suggesting the promoter strength had an important impact on production. Furthermore, the increased transcription levels of the biosynthetic genes *tcsA*, *fkbG*, *fkbB* and *fkbO*, which were located in different transcription units*,* were also detected in strain L19-9 by qRT-PCR (Fig. 6b).

### Production-stability of the hyper-production strain L19-9

In industrial production, production-degeneration of the strain is a common problem and the continuous screening process is always laborious and time-consuming. Genetic stability of the hyper-production strain L19-9 was then investigated by continuous passage on ISP4 agar plates. After continuously culturing for 5 generations, FK506 production of each generation was analyzed at 168 h, respectively. The results showed that the production of each generation hardly changed, indicating the production-stability and the potential for industrialization of the high-yielding strain L19-9 (Fig. 6c).

### Further improvement of FK506 production by L-isoleucine addition

**Fig. 7:**
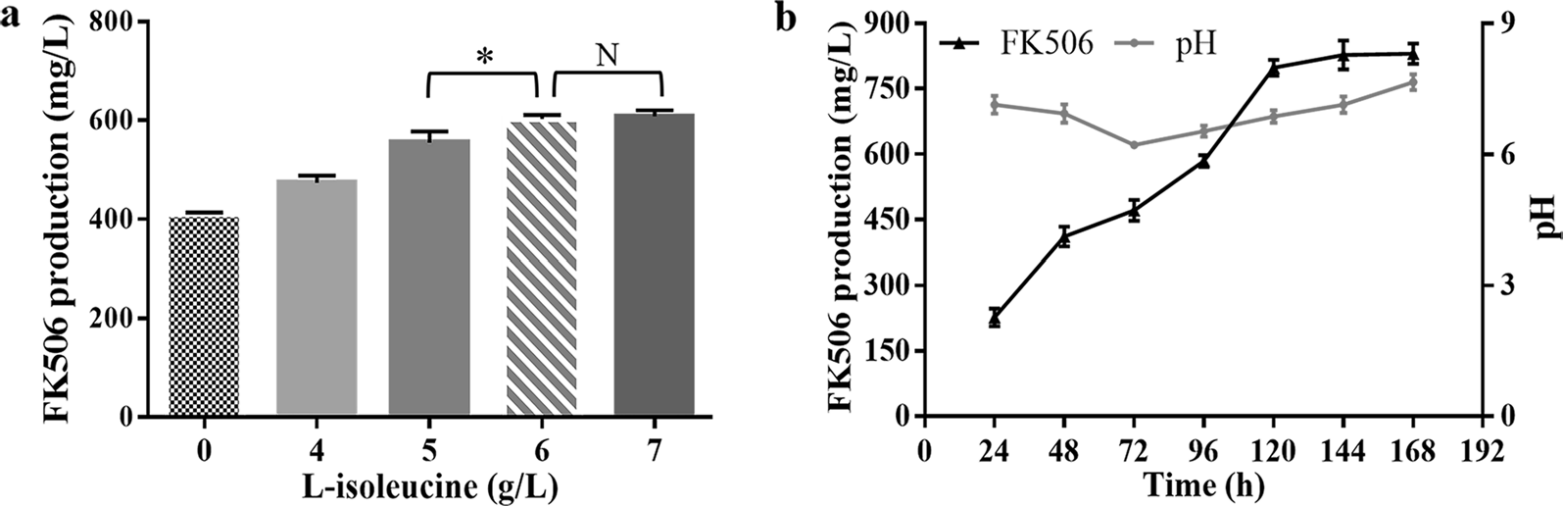
Effect of L-isoleucine supplement on FK506 production. **a** Effect of different concentrations of L-isoleucine on FK506 production in shake-flask culture. **b** FK506 production analyzed in a 15-L stirred-tank bioreactor with 8-L Fm_1_. Error bars show standard deviations of three independent experiments. The asterisks indicated the statistic significant differences. *P < 0.05; N, no significant difference

It has been previously reported that FK506 production was closely related with primary metabolism and could be improved by genetic manipulation of related genes [20]. Accordingly, related genes including *accA2B/E*, *dahp*, *gdhA* and *zwf2* was genetically manipulated individually in strain L19-9 and the effect of the above-mentioned targets on FK506 production was then investigated. However, none of the targets showed obvious effect on FK506 production (data not shown), implying that the already known targets might not be applicable to other strains due to specific genomic contexts and culture conditions. Thus, the medium-optimization for strain L19-9 was then conducted to increase FK506 yield in shake-flask culture. The effects of various nutrients (including glycerol, methyl oleate, L-lysine and L-isoleucine) on FK506 production were examined by individually adding each component to the Fm medium at a final concentration of 5 g/L. Among the tested components, only L-isoleucine showed positive effect on FK506 production and different concentrations of L-isoleucine were also evaluated to determine the optimum amount added to the Fm medium. The results demonstrated that a final concentration of 6 g/L was optimal, with FK506 production increased from 410.3 to 603 mg/L (Fig. 7a).

To further evaluate the FK506 production capacity for strain L19-9 in the optimized medium, scale-up fermentation was then carried out in a 15-L stirred-tank bioreactor with 8-L Fm_1_ medium (adding 6 g/L of L-isoleucine into Fm medium). The aeration was kept at 1.0 vvm (air/culture volume/min) and the agitation rate was set around 150–250 rpm to maintain dissolved oxygen levels around 35% during the culture process. It was observed that FK506 production of the strain L19-9 in scale-up fermentation could reach 830.3 mg/L (Fig. 7b).

## Discussion

To date, tremendous efforts have been made to enhance FK506 production. For example, FK506 production was dramatically increased by combinational strategies (such as random mutation, biosynthetic genes overexpression and exogenous feeding) and two high-yielding strains were constructed, with final FK506 production of 429 mg/L and 457 mg/L, respectively [16, 34]. Basing on optimization of media and culture conditions in *S. tsukubaensis* NBRC 108819, FK506 production could be increased from 205 ± 22.0 to 616 ± 52.2 mg/L in a 2.5-L stirred-tank bioreactor [35]. However, there were few studies of an FK506 over-producer constructed rationally and systematically by taking both hyper-production and industrial application into consideration. In present work, a strategy for hyper-production and industrial application of FK506 was developed and FK506 production was increased from 140.3 to 830.3 mg/L.

The chassis of *Streptomyces* refers to the engineered strains suitable for hetero-expression, in which the energy and precursor pool was improved to increase production of desired products [36, 37]. For production-improvement of known products in its native strain, some strategies used in the construction of chassis can also be taken into consideration. Each *Streptomyces* contains a wide range of biosynthetic gene clusters [38, 39] and deletion of the putatively competitive pathways might increase the production of desired products [40, 41].Inspired by these studies, biosynthetic gene clusters (mainly PKS/PKS-NRPS gene clusters) in *S. tsukubaensis* L19 were also analyzed and core PKS located in transcription-active gene clusters were deleted, creating a better strain L19-2 with a cleaner metabolic background and making the FK506 production increased by 21.4%.

The detailed understanding of a targeted biosynthetic pathways was essential for rational and systematical engineering. For example, the production of jadomycin B and novobiocin was improved by substitution of the native promoters of related biosynthetic genes with stronger constitutive and inducible promoters, respectively [42, 43]. In our previous work, the FK506 biosynthetic gene cluster, transcription units within this cluster and its pathway-specific regulatory mechanism were illustrated [17]. In strain L19-2, we then focused on refactoring the FK506 biosynthetic gene cluster to further increase FK506 production. For this purpose, putative rate-limiting steps were first analyzed by either overexpression of biosynthesis-related genes or in-situ substitution of the promoter regions. Unexpectedly, no rate-limiting step was found and the FK506 production even decreased by substituting promoters of the core biosynthetic genes with the constructed promoter cassettes *Pke* or *Pgr*. This phenomenon might be explained by the fact that balanced metabolism between cell growth and product formation is critical and the insertion of strong constitutive promoters upstream of biosynthetic genes may cause metabolic disorders [44–46]. The unexpected results indicated that the promoter substitution strategy might vary in different strains and how to enhance the transcription levels of the entire FK506 biosynthetic gene cluster in a precise and fine-tuning manner was crucial. Herein, as proof of this idea, the promoter substitution strategy was subsequently conducted to manipulate expression of the regulatory genes *fkbN* and *tcs7*, which successfully and dramatically enhanced expression of the FK506 biosynthetic gene cluster and production of FK506. Furthermore, we also tried to introduce another copy of the regulatory genes (*fkbN* and *tcs7*) into strain L19-9, which lead no further improvement of FK506 production. It should be noted that strain L19-9 derived from the in-situ promoter substitution strategy had higher FK506 production and was more conducive for further genetic manipulation and potential industrialization, since only one selectable marker (apramycin resistance gene) could be utilized in this strain and no additional resistance gene was introduced by this in-situ substitution strategy.

Considering that FK506 is produced from common building blocks deriving from primary metabolic pathways, we also evaluated the effects of genes related to primary metabolism and nutrients-addition on FK506 production. By adding L-isoleucine at a final concentration of 6 g/L, the FK506 production was further increased from 410.3 to 603 mg/L in shake-flask culture, and the production could reach the highest of 830.3 mg/L by scale-up fermentation. Previous studies indicated that L-isoleucine could be transformed into acetyl-CoA, succinyl-CoA and propionyl-CoA, enhancing both primary metabolism and precursors for FK506 biosynthesis [16, 25, 47, 48]. With the guidance of various omics information, future study may focus on identifying dominant gene targets relating to both L-isoleucine-addition and FK506 production, which could be further manipulated to maximize the potential of FK506 production in the industrial strain L19-9.

## Conclusions

**Fig. 8:**
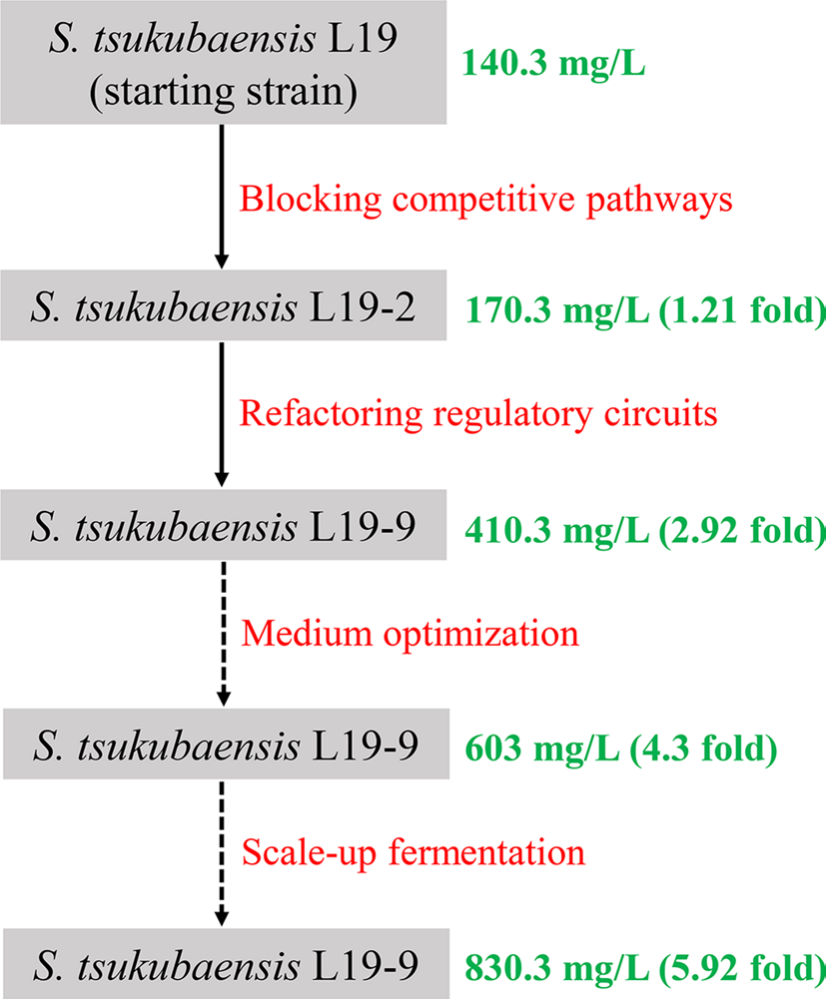
Related manipulations and FK506 production of the engineered strains constructed in this study

In this work, basing on specific features of the FK506-producing strain L19 and taking both hyper-production and industrial application into consideration, the FK506 production was rationally and systematically improved from the aspects of competitive pathways, biosynthetic gene cluster refactoring and medium-optimization (Fig. 8). It should be noted that, if necessary, the FK506 production in strain L19-9 could be optimized repeatedly by deeper investigation. Using the same experimental set-up, this strategy presented here can in principle enable the hyper-production of other valuable natural products or FK506 in other *Streptomyces* strains.

## Methods

### Bacterial strains and culture conditions

Strains used in this work were listed in Additional file 2: Table S1. *E. coli* TG1 was used as host for plasmid cloning. *E. coli* ET12567/pUZ8002 was used for conjugation to transfer plasmids into *Streptomyces* strains. *E. coli* was cultured in LB liquid medium (1% w/v tryptone, 0.5% w/v yeast extract and 1% w/v NaCl) or on LB agar plates at 37 °C. All *Streptomyces* strains were grown on ISP4 solid medium (BD, USA) for spore preparation or conjugation and in TSB (3% trypticase soy broth, w/v) for preparation of genomic DNA or seed medium. The Fm medium (5% w/v maltodextrin, 1% w/v yeast power, 3% w/v cotton seed meal, 0.2% w/v K_2_HPO_4_, 0.1% w/v CaCO_3_ and pH 7.0) and Fm_1_ medium (adding 6 g/L of L-isoleucine into Fm) were used as fermentation medium for production of FK506. For culturing *E. coli* ET12567/pUZ8002 carrying related constructed plasmids used for intergeneric conjugation, antibiotics were supplemented to growth media at the following final concentrations: kanamycin, 50 μg/mL; apramycin, 50 μg/mL; chloramphenicol, 25 μg/mL. For intergeneric conjugation, after co-culturing for about 18 h, apramycin and nalidixic acid were coated on ISP4 agar plates at the final concentration of 50 and 25 μg/mL, respectively.

### Plasmid construction

Plasmids and primers used in this work were listed in Additional file 2: Tables S1 and S2, respectively. The genome sequence of *S. tsukubaensis* has been deposited in GenBank (accession number CP070379). The gene cassettes *tcsA/B/C/D* and *fkbO* were amplified from the genomic DNA using primer pairs 1 and 2, respectively. The fragments were then individually cloned into *Nde*I/*Not*I-digested pLM1 [28] using the ClonExpress II one-step cloning kit (Vazyme biotech, Nanjing, China) and confirmed by sequencing, yielding plasmids (pLM1-1 and pLM1-3) for gene overexpression. The gene cassettes *fkbH/I/J/K* and *fkbG* were amplified using primer pairs 3 and 4, respectively. The fragments were then cloned into *Nde*I/*Not*I-digested pLM1 to yield pLM1-2. For construction of bidirectional promoter cassettes *Pke* and *Pgr*, the ermEp* was amplified from pLM1 using primer pairs 5. The other promoters (*gapdhp*, kasOp*and *rpsLp*) were synthesized according to reported literature [29, 31] and amplified using primer pairs 6, 7 and 8, respectively. The ermEp* and *gapdhp* were then individually cloned into *Bam*HI-digested pSET152 [49] using the above cloning kit. The resultant plasmids were digested with *Eco*R**V**, and kasOp*and *rpsLp* were then individually cloned into the site, yielding plasmids pSET152-KE and pSET152-GR containing bidirectional promoter cassettes *Pgr* and *Pke*, respectively*.*

For deletion of the intergenic region between the gene *fkbB* and *fkbO*, two 1.5-kb DNA fragments flanking the region were amplified from the genomic DNA of *S. tsukubaensis* L19 using primer pairs 9 and 10, respectively, and then cloned into *Eco*RI/*Hin*dIII-digested pKC1139 [49] generating the disruption plasmid pKC1139-ΔBO. Accordingly, the disruption plasmids pKC1139-C3, pKC1139-C6 and pKC1139-C9 were also constructed as mentioned above, using primer pairs 11, 12, 13, 14, 15 and 16.

For promoter substitution of the intergenic region between the gene *fkbB* and *fkbO*, two 1.5-kb DNA fragments flanking the region were amplified using primer pairs 17 and 18. The bidirectional promoter cassette *Pke* was amplified from pSET152-KE using primer pairs 19 and 20. These three fragments were then cloned into *Eco*RI/*Hin*dIII-digested pKC1139 generating the plasmid pKC1139-ΔBOKE. Accordingly, other plasmids pKC1139-ΔBOGR, pKC1139-ΔGR and pKC1139-ΔKE used for in-situ promoter-substitution were also constructed.

### Construction of *S. tsukubaensis* strains

The overexpression plasmids were transformed into *E. coli* ET12567/pUZ8002 and then introduced into *S. tsukubaensis* L19-2 via intergeneric conjugation. To get the over-expression strains, exconjugants were selected on ISP4 agar plates supplemented with apramycin and identified by PCR as described previously [17].

For gene deletion or in-situ substitution, the plasmids derived from pKC1139 were introduced into *S. tsukubaensis* by conjugation as mentioned above. Single-crossover-recombination strains were selected by culturing the transformants on ISP4 plates containing 50 μg/mL apramycin at 37 °C. Subsequently, after two rounds of sporulation on plates without antibiotics at 28 °C, double-crossover mutants were selected by their apramycin sensitivity and confirmed by PCR.

### RNA extraction and quantitative real-time PCR (qRT-PCR)

The RNA of *S. tsukubaensis* strains was extracted from mycelia cultured in Fm medium for 36 h, using EASYspin Plus bacteria RNA extract kit (Aidlab Biotech, Beijing, China) according to the manufacturer’s instructions. Residual genomic DNA was digested with RNase-free DNase I (TaKaRa, Tokyo, Japan). The cDNA was synthesized using M-MLV reverse transcriptase (TaKaRa, Tokyo, Japan) according to the manufacturer’s instructions.

To analyze transcription of the 10 PKS/PKS-NRPS gene clusters in L19, the expression of their core PKS was detected by RT-PCR with the primer pairs described in Additional file 2: Table S2. Quantitative real-time polymerase chain reaction (qRT-PCR) was performed using SYBR PremixEx Taq II (TaKaRa, Tokyo, Japan) as described previously [36, 50]. The sigma factor gene *hrdB* was used as an internal control to normalize the transcriptional levels. The fold changes of the transcriptional levels were calculated by the ${2}^{{ - \Delta \Delta {\text{C}}_{{\text{t}}} }}$ method as described previously [51]. The software GraphPad Prism (version 6.02) was used to analyze qRT-PCR data. To compare the difference between the test and control data, *P* values were calculated by Student’s *t* test. Each experiment was performed in triplicate. PCR primers used here were listed in Additional file 2: Table S2.

### Fermentation and analysis of FK506 and packed mycelium volume (PMV)

The strains were cultured on ISP4 agar plates for about 7–10 days at 28 °C for sporulation. The spores were then inoculated into 25-mL TSB medium (seed medium) in 250-mL flasks and cultured at 28 °C and 220 rpm for 24 h. For shake-flask fermentation, the seed culture was inoculated into 30-mL fermentation medium giving a final OD600 of 0.4, and then cultured at 28 °C and 220 rpm for 168 h. For fermentation in a 15-L stirred-tank bioreactor, the seed culture (400 mL)was then transferred into the fermenter containing fermentation medium (8 L). The aeration was kept at 1.0 vvm (air/culture volume/min) and the agitation rate was set around 150–250 rpm to maintain dissolved oxygen levels around 35% during the culture process.

For analysis of FK506, the 200 µL culture sample from Fm was ultrasonically extracted with 2.5-fold volume of methanol. The supernatant was then recovered by centrifugation at 12,000 rpm for 10 min and injected into a HPLC system (Agilent, Palo Alto, CA, USA) equipped with an Eclipse Plus C18 column (5 mm, ⌀ 4.6 × 150 mm), following the program with solvent A (0.1% phosphoric acid in water) and solvent B (acetonitrile): 0–25 min, 68% A/32% B. The UV detection was set at 210 nm. The flow rate was 1 mL/min and the column temperature was 60 °C. For calculation of packed mycelium volume (PMV), the culture broth (1 mL) was centrifuged at 4,000 × *g* for 4 min. The PMV was then calculated by dividing the volume of packed mycelium by the total volume of the culture broth (1 mL). The software GraphPad Prism (version 6.02) was used to analyze the data. To compare the difference, *P* values were calculated by Student’s *t* test. Each experiment was performed in triplicate.

## Supplementary Information

**Additional file 1**: **Fig. S1**. Analysis of expression of related gene clusters in strain L19 by RT-PCR. Genome DNA (g); complementary DNA (c).

**Additional file 2**: **Table S1**. Strains and plasmids used in this work. **Table S2**. Primers used in this work.

## Data Availability

The datasets used and/or analyzed during the current study are available from the corresponding author on reasonable request.

## Abbreviations

PKS: Polyketide synthase
NRPS: Non-ribosomal peptide synthase
RT-PCR: Reverse transcription polymerase chain reaction
qRT-PCR: Quantitative real-time polymerase chain reaction
HPLC: High-performance liquid chromatography
