# Supplementary material for: Improvement of FK506 production via metabolic engineering-guided combinational strategies in Streptomyces tsukubaensis
Source: Microb Cell Fact. 2021 Aug 23;20:166. doi: 10.1186/s12934-021-01660-w (PMC8383387; doi:10.1186/s12934-021-01660-w)
Supplement: Supplementary file 1 — Additional file 1: Fig. S1. Analysis of expression of related gene clusters in strain L19 by RT-PCR. Genome DNA (g); complementary DNA (c). [file 12934_2021_1660_MOESM1_ESM.docx]

**
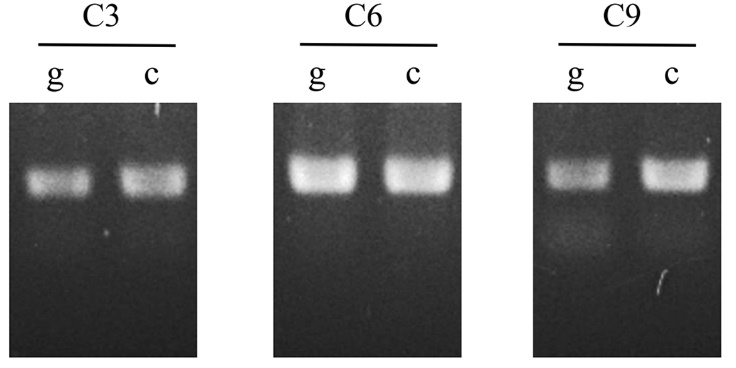
**

**Fig. S1 Analysis of expression of related gene clusters in strain L19 by RT-PCR.** Genome DNA (g); complementary DNA (c).
