# Supplementary material for: Improvement of FK506 production via metabolic engineering-guided combinational strategies in Streptomyces tsukubaensis
Source: Microb Cell Fact. 2021 Aug 23;20:166. doi: 10.1186/s12934-021-01660-w (PMC8383387; doi:10.1186/s12934-021-01660-w)
Supplement: Supplementary file 2 — Additional file 2: Table S1. Strains and plasmids used in this work. Table S2. Primers used in this work. [file 12934_2021_1660_MOESM2_ESM.docx]

**Table S1 Strains and plasmids used in this study.**

| **Strains or plasmids** | **Description** | **Reference** |
| --- | --- | --- |
| **Strains** |  |  |
| *S. tsukubaensis* L19 | Wild type FK506 producer | This study |
| *S. tsukubaensis* L19-1 | Strain abolished the FK506 production | This study |
| *S. tsukubaensis* L19-2 | Blocking putative competitive pathways (C3, C6 and C9) in L19 | This study |
| *S. tsukubaensis* L19-3 | The gene cassettes *tcsA/B/C/D* overexpressed in L19-2 | This study |
| *S. tsukubaensis* L19-4 | The gene cassettes *fkbG/H/I/J/K* overexpressed in L19-2 | This study |
| *S. tsukubaensis* L19-5 | The gene cassettes *fkbO* overexpressed in L19-2 | This study |
| *S. tsukubaensis* L19-6 | Intergenic region between *fkbB* and *fkbO* replaced by *Pke* | This study |
| *S. tsukubaensis* L19-7 | Intergenic region between *fkbB* and *fkbO* replaced by *Pgr* | This study |
| *S. tsukubaensis* L19-8 | Intergenic region between *tcs6* and *tcs7* replaced by *Pke* | This study |
| *S. tsukubaensis* L19-9 | Intergenic region between *tcs6* and *tcs7* replaced by *Pgr* | This study |
| *E. coli* TG1 | General cloning strain | Novagen, China |
| ET12567/pUZ8002 | Methylation-deficient strain for conjugation | FengBio, China |
| **Plasmids** |  |  |
| pLM1 | Integrative shuttle vector, *ermEp**, *apra*^R^ | [1] |
| pLM1-1 | Plasmid deriving from pLM1 used for *tcsA/B/C/D* overexpression | This study |
| pLM1-2 | Plasmid deriving from pLM1 used for *fkbG/H/I/J/K* overexpression | This study |
| pLM1-3 | Plasmid deriving from pLM1 used for *fkbO* overexpression | This study |
| pSET152 | Integrative shuttle vector | [2] |
| pSET152-KE | pSET152 containing bidirectional promoter cassette *Pke* | This study |
| pSET152-GR | pSET152 containing bidirectional promoter cassette *Pgr* | This study |
| pKC1139 | Vector for gene disruption in *Streptomyces* | [2] |
| pKC1139-ΔBO | Plasmid used for abolishing FK506 production | This study |
| pKC1139-C3 | For deletion of core biosynthetic gene in cluster C3 | This study |
| pKC1139-C6 | For deletion of core biosynthetic gene in cluster C6 | This study |
| pKC1139-C9 | For deletion of core biosynthetic gene in cluster C9 | This study |
| pKC1139-ΔBOGR | For substitution of the intergenic region between the gene *fkbB* and *fkbO using Pgr* | This study |
| pKC1139-ΔBOKE | For substitution of the intergenic region between the gene *fkbB* and *fkbO using Pke* | This study |
| pKC1139-ΔGR | For substitution of the intergenic region between the gene *tcs6* and *tcs7* using *Pgr* | This study |
| pKC1139-ΔKE | For substitution of the intergenic region between the gene *tcs6* and *tcs7* using *Pke* | This study |

1. Mao XM, Zhou Z, Cheng LY, Hou XP, Guan WJ, Li YQ. Involvement of SigT and RstA in the differentiation of Streptomyces coelicolor. FEBS Lett*.* 2009;583:3145-3150.

2. Bierman M, Logan R, O'Brien K, Seno ET, Rao RN, Schoner BE. Plasmid cloning vectors for the conjugal transfer of DNA from Escherichia coli to Streptomyces spp. Gene*.* 1992;116:43-49.

**Table S2 Primers used in this study.**

| Primer pairs | Primer | Sequence (5’-3’) ^[a]^ | Description |
| --- | --- | --- | --- |
| 1 | TcsAD F | ACCAAAGGAGGCGGACATATGACCAGTGGGGTGGCGTTCCT | Amplification of gene *tcsA/B/C/D* |
|  | TcsAD R | CCAAGATCTGCCCAGCACGCGGGACTGACCAGAACAT |  |
| 2 | FkbO F | ACCAAAGGAGGCGGACATATGGTGCCTGTCGCGGCACCGT | Amplification of gene *fkbO* |
|  | FkbO R | CCAAGATCTGCCCAGCATCACGACACCACTCCTTCG |  |
| 3 | FkbHK F | TGTTGCGGAAGCGGTGACAAAGGAGGCGGACATATGGTGACGCCGGCCGGGGGAAG | Amplification of *fkbH/I/J/K* |
|  | FkbHK R | CCAAGATCTGCCCAGCATCACCTTGTTGCGGAAGCGG |  |
| 4 | FkbG F | ACCAAAGGAGGCGGACATATGgcgaatcaggtgaccatgtc | Amplification of gene *fkbG* |
|  | FkbG R | TCACCGCTTCCGCAACAAGG |  |
| 5 | ermEp F | GCGGTCGACTCTAGAGTGTCCGCCTCCTTTGGTCAC | Amplification of *ermEp** |
|  | ermEp R | ATCGCGCGCGGCCGCGGGCGGCTTGCGCCCGATGCT |  |
| 6 | Gapdhp F | GCGGTCGACTCTAGAGGCGTATCCCCTTTCAGATAC | Amplification of *gapdhp* |
|  | Gapdhp R | ATCGCGCGCGGCCGCGGCTGCTCCTTCGGTCGGACG |  |
| 7 | KasOp F | CGCGGCCGCGCGCGATTGTTCACATTCGAACGGTCT | Amplification of *kasOp** |
|  | KasOp R | GATTACGAATTCGATAACTCCCCCAGTCCTGCACG |  |
| 8 | Rpsl F | CGCGGCCGCGCGCGATGCCCTGCAGGCGGAAGTCAG | Amplification of *rpsLp* |
|  | Rpsl R | GATTACGAATTCGATTACGTCTCCGTCGTCTACT |  |
| 9 | BO-L-F | CGACGGCCAGTGCCAccgagtagtgggctgcgag | Amplification of homologous left arm |
|  | BO-L-R | cagcaattcgtgcacgtgcctgtcgcggcaccgt |  |
| 10 | BO-R-F | gtgcacgaattgctgcgcgt | Amplification of homologous right arm |
|  | BO-R-R | TATGACATGATTACGtgagcgcctcgtcccagag |  |
| 11 | C3-L-F | AAACGACGGCCAGTGCCAGTCCCTGTTCATGCACGGCAACC | Amplification of homologous left arm |
|  | C3-L-R | GAGGCGGAGACGGCAACGTAGT |  |
| 12 | C3-R-F | GTTGCCGTCTCCGCCTCGTGCCGGTGACCGCCCCCAAGAC | Amplification of homologous right arm |
|  | C3-R-R | AGCTATGACATGATTACGGTGTCAGTCGCTCTGCCAGGAGT |  |
| 13 | C6-L-F | AACGACGGCCAGTGCCAGGAGCACGCTGTTGAGGTTGG | Amplification of homologous left arm |
|  | C6-L-R | CCGCCCACCGCCAATCTGCAC |  |
| 14 | C6-R-F | AGATTGGCGGTGGGCGGGATGCCGGTGATGACGACAC | Amplification of homologous right arm |
|  | C6-R-R | GCTATGACATGATTACGCGAAGAATCCGACCCGTTCAC |  |
| 15 | C9-L-F | AACGACGGCCAGTGCCACGTCCTCAACACCCTCCTCCTG | Amplification of homologous left arm |
|  | C9-L-R | GTCGGGTGCCTTGGGAAGAC |  |
| 16 | C9-R-F | CTTCCCAAGGCACCCGACTGAGGCATCGGCCATCTGGAAGGC | Amplification of homologous right arm |
|  | C9-R-R | GCTATGACATGATTACGTGGGTACGGACCAGCTCAAGG |  |
| 17 | BO-S LF | CGACGGCCAGTGCCAccgagtagtgggctgcgag | Amplification of homologous left arm for substitution |
|  | BO-S LR | gtgcctgtcgcggcaccgt |  |
| 18 | BO-S RF | gtgcacgaattgctgcgcgt | Amplification of homologous right arm substitution |
|  | BO-S RR | TATGACATGATTACGtgagcgcctcgtcccagag |  |
| 19 | Pke-BOF | cagcaattcgtgcactgtccgcctcctttggtcac | Amplification of *Pke* for substitution of the region between *fkbB* and *fkbO* |
|  | Pke-BOR | TgccgcgacaggcacAACTCCCCCAGTCCTGCACG |  |
| 20 | Pgr-BOF | cagcaattcgtgcacGCGTATCCCCTTTCAGATAC | Amplification of *Pgr* for substitution of the region between *fkbB* and *fkbO* |
|  | Pgr-BOR | TgccgcgacaggcacTACGTCTCCGTCGTCTACTC |  |
| 21 | Tcs67S-LF | AAACGACGGCCAGTGCCACGACACCTTCGCCCACTACCG | Amplification of homologous left arm for substitution |
|  | Tcs67S-LF | GTGAGACTCCCCGGCGAGAT |  |
| 22 | Tcs67S-RF | ATGTTTCGACGTTTTTCTGC | Amplification of homologous right arm substitution |
|  | Tcs67S-RF | TATGACATGATTACGGTGAGACTCCCCGGCGAGATG |  |
| 23 | Pke-67F | gccggggagtctcactgtccgcctcctttggtcac | Amplification of *Pke* for substitution of the region between *fkbN* and *tcs7* |
|  | Pke-67R | gaaaaacgtcgaaacatAACTCCCCCAGTCCTGCACG |  |
| 24 | Pgr-67F | gccggggagtctcacGCGTATCCCCTTTCAGATAC | Amplification of *Pgr* for substitution of the region between *fkbN* and *tcs7* |
|  | Pgr-67R | gaaaaacgtcgaaacatTACGTCTCCGTCGTCTACTC |  |
| 25 | TcsA F | GCGCTTCTTCTACTCCCA | qRT-PCR primer for *tcsA* |
|  | TcsA R | TCATGTCGTTCACCATCAG |  |
| 26 | FkbG F | GTCGTTCGATGTCGTGTT | qRT-PCR primer for *fkbG* |
|  | FkbG R | CTCGTAGTAGGCCGGATAG |  |
| 27 | FkbB F | ACAGCGGGCTACCTCTAC | qRT-PCR primer for *fkbB* |
|  | FkbB R | GTCGTCCTCCTGTTCTCC |  |
| 28 | FkbO F | GTTTCTGCACACCGACAT | qRT-PCR primer for *fkbO* |
|  | FkbO R | TCACGACACCACTCCTTC |  |
| 29 | FkbQ F | GGCGTTGATTAACAGCAT | qRT-PCR primer for *fkbQ* |
|  | FkbQ R | GACCGTGGAGGTCTTCTC |  |
| 30 | C3-F | TCACCGGCATCGGGGTCGT | RT-PCR primer for cluster C3 |
|  | C3-R | GTTCGAAATCGCGGACCTC |  |
| 31 | C6-F | GATGGAGCTGGGCACGAAGT | RT-PCR primer for cluster C6 |
|  | C6-R | CCGGACCGGTGTCACCATC |  |
| 32 | C9-F | CACTCCCCAGGGCCGGGGTG | RT-PCR primer for cluster C9 |
|  | C9-R | CGGCGGGGATATCGGTGACG |  |
| 33 | orf7063F | GCAGCACTCGACGCGCTGC | qRT-PCR primer for *orf7063* |
|  | orf7063R | TCACCGCTTCCGCAACAAGG |  |
| 34 | orf7031F | GCTGCTCGACGCCGCCGAC | qRT-PCR primer for *orf7031* |
|  | orf7031R | CGTGGTGTGGCCGCCGAAC |  |
